# Evaluation of the effect of eltrombopag therapy on the platelet collagen receptor glycoprotein VI (GPVI) expression and soluble GPVI levels in young patients with immune thrombocytopenia

**DOI:** 10.1007/s11239-022-02734-1

**Published:** 2022-12-07

**Authors:** Azza Abdel Gawad Tantawy, Nayera Hazaa Khalil Elsherif, Fatma Soliman Ebeid, Rasha Abd El-Rahman El-Gamal, Eman Abdel Rahman Ismail, Mahmoud A. Kenny, Michael Botros Elkes morcos

**Affiliations:** 1 Pediatrics Department, Faculty of Medicine, Ain Shams University, Cairo, Egypt; 2 Clinical Pathology Department, Faculty of Medicine, Ain Shams University, Cairo, Egypt; 3 Abbaseya square, Eldemerdash Hospital, cairo, Egypt

**Keywords:** Chronic ITP, Eltrombopag, GPV1 receptor expression, Soluble GPV1

## Abstract

**Background:**

Platelet glycoprotein VI (GPVI) receptor is essential for platelet adhesion and aggregation. Eltrombopag is as an effective treatment for chronic immune thrombocytopenia (ITP); yet, its effect on platelet function is not fully characterized.

**Aim:**

This prospective study investigated the effect of eltrombopag therapy on platelet function through assessment of GPVI receptor expression and soluble GPVI levels among pediatric patients with persistent or chronic ITP.

**Methods:**

Thirty-six children and adolescents with persistent or chronic ITP were divided equally into two groups either to receive eltrombopag therapy or the standard of care. All patients were followed-up for 12 months with assessment of bleeding score and complete blood count (CBC). Evaluation of GPVI expression using flow cytometry and measurement of its soluble form by ELISA was done at baseline and at 6 months.

**Results:**

ITP patients on eltrombopag had significantly lower bleeding score after 6 months of therapy while the quality of life has significantly improved. Platelet count was significantly increased throughout the study. GPVI expression by flow cytometry and soluble GPVI levels were significantly increased after eltrombopag therapy. After 12 months, ITP patients on eltrombopag were able to maintain a good quality of life and low bleeding score.

**Conclusion:**

Our data suggest that eltrombopag, through its effect on the GPVI receptor expression and its soluble form, might reduce bleeding manifestations and improve the quality of life of chronic and persistent ITP children independent of its effect on the platelet count.

## Introduction

The presentation of immune thrombocytopenia (ITP) may be dramatic, with extensive soft tissue bruising and mucosal hemorrhage, but it is more often a self-limiting condition [1]. The concern for bleeding in ITP is greatest at platelet counts less than 20 to 30 × 10^9^/L. Bleeding typically occurs in the skin or mucous membranes in a ‘platelet-type’ pattern, with petechiae, bruising, epistaxis and gum bleeding being common. Severe bleeding manifestations such as intracranial hemorrhage, gastrointestinal or genitourinary bleeding and severe menstrual bleeding are relatively uncommon [2, 3].

Novel thrombopoiesis-stimulating agents have been developed to increase platelet production and thereby, correcting thrombocytopenia in patients with ITP [4]. These compounds called thrombopoietin (TPO) receptor agonists (TPO-RAs) and show no structural resemblance to TPO but still bind and activate the TPO receptor (TPO-R) [5]. Eltrombopag is an oral, non-peptide, TPO-RA that stimulates thrombopoiesis, leading to increased platelet production. Eltrombopag has been reported to be an effective treatment for management of thrombocytopenia in adults with chronic ITP [6, 7].

Patients with ITP often have few bleeding symptoms despite very low platelet counts, suggesting that platelets may be functional [8]. Although TPO stimulation in vitro does not directly activate platelets, it potentiates platelet reactivity to several agonists, including adenosine diphosphate (ADP), thrombin, and collagen [9]. However, the effect of eltrombopag on platelet function in vivo in thrombocytopenic patients is still debatable. The hypothesis of its effect on platelet activation and its potential for thrombosis with normal platelet counts is suggested through either direct stimulation of platelet TPO receptors rendering them more susceptible to lower concentrations of agonists or indirectly by the influx of new potentially more reactive platelets [10].

Glycoprotein (GP) PIbα of the GPIb-IX-V complex and GPVI are of particular interest because these receptors are essentially platelet specific. They are critical for initiation of thrombus formation at arterial shear rates and are implicated in wider platelet functions beyond hemostasis and thrombosis, as well as platelet aging and clearance [11]. GPVI is thought to be the major signaling receptor involved in platelet activation on exposed collagen. Following GPVI interactions with collagen, platelets initiate strong activation and release the content of α- and dense granules [12].

Published data suggested that GPVI expression was up-regulated in megakaryocytes after TPO stimulation. [13, 14]. There is a proposed hypothesis that eltrombopag can up-regulate GPVI expression in ITP patients and thereby, enhancing platelet adhesion leading to improvement of bleeding profile of those patients [15]. Therefore, we investigated the effect of eltrombopag therapy on platelet function through the assessment of GPVI receptor expression and soluble GPVI levels among pediatric patients with persistent or chronic ITP.

## Materials and methods

This prospective study included children and adolescents with chronic and persistent ITP aged ≤ 18 years. Patients having a bleeding score grade 4 or more using ITP Bleeding Scale (IBLS) at the baseline visit [16] were recruited from the regular attendants of the Pediatric Hematology Clinic, Pediatrics Hospital, Ain Shams University. Patients with acute ITP, Evans syndrome, or abnormal liver and kidney function tests were excluded. The study protocol was approved by the local ethical committee of Ain Shams University and in accordance with Helsinki Declaration of 2008. Consent forms were obtained from the patients or their legal guardians after they were informed about the study and its expected outcomes. Reporting of the study conforms to Consolidated Standards of Reporting Trials (CONSORT) 2010 statement [17].

### Sample size

Sample size was determined using PASS program, setting alpha error 5% and Power 80%. Results from a previous study [18] showed that the improvement in platelet count was 59% among eltrombopag group compared to 16% among control group. Based on this, the needed sample is 15 patients per group which was raised to 18 considering a 10% drop-out rate and therefore, the total sample size was 36 patients with persistent or chronic ITP.

### Study groups

A total of 36 patients who had either persistent ITP (defined as ongoing ITP between 3 and 12 months from diagnosis) or chronic ITP (defined as ITP lasting more than 12 months) [19, 20] with partial or no response to first line of therapy were enrolled and randomly assigned into one of two groups;

Group 1 (n = 18): Patients received a total daily dose of eltrombopag of (25-75 mg/d); for pediatric patients from 1 to 5 years, the starting dose was 25 mg/d and for pediatric patients ≥ 6 years, the starting dose was 50 mg/d. Dose adjustment was made based on platelet count with an increment of 25 mg once per day at 2 weeks intervals (Maximum dose: 75 mg orally once a day) [21, 22]. If platelet count reached more than 200 × 10^9^/L, the dose was decreased by 25 mg once per day at 2 weeks intervals. If platelet count increased to more than 400 × 10^9^/L, treatment was interrupted until platelet counts decreases to less than 150 × 10^9^/L then the dose was resumed at the next lower dose. Group 2 (n = 18): Patients who received other lines of treatment (steroids, intravenous immunoglobulins (IVIG), or immunosuppressive drugs) served as controls. Twenty age- and sex-matched healthy children were enrolled as healthy controls to assess reference GPVI receptor expression and soluble GPVI levels.

All included patients were subjected to detailed medical history and clinical examination for signs and type of bleeding and anthropometric measures. Data were collected from the patients’ medical files focusing on age at diagnosis, disease duration and number of previous lines of treatments. Assessment of the bleeding score was performed at baseline and repeated at 6 months as well as at the end of the study [16]. The health-related quality of life using Kids’ ITP Tools (KIT) questionnaires was completed and evaluated at baseline and 6 months [23].

Laboratory analysis included baseline and every 3 months complete blood count (CBC) using Sysmex XT-1800i (Sysmex, Kobe, Japan), liver function tests, bone marrow examination with reticulin stain, assessment of GPVI expression using flow cytometry and soluble GPVI levels using enzyme linked immunosorbent assay (ELISA) at baseline and at 6 months.

### Assessment of GPVI receptor expression and soluble form

The surface expression of the platelet receptors GPVI was measured by flow cytometry [24, 25] with the use of the following monoclonal antibodies: fluorescein isothiocyanate (FITC)-labeled anti-CD41 (Beckman Coulter, Inc., Fullerton, CA, USA) and phycoerythrin (PE)-labeled mouse anti-Human platelet GPVI (Cat. No. 565,241; BD Pharmingen™, CA, USA). Processed samples were delivered to the lab and stained with monoclonal antibodies within 30 min of collection to avoid platelet adhesion or aggregation.

In brief, a volume of 50 µL of blood was added to 5 µL of fluorochrome-labeled monoclonal antibody. Data were analyzed using Navios flow cytometer (2-laser, 6-color) (Beckman Coulter, Inc., Hialeah, FL, USA). Measurements and analysis were performed using Navios software version 1.1. The CD41-positive platelets were gated and the expression of GPVI-PE was evaluated. Each measurement was performed in duplicate and median fluorescence intensity (MFI) was used to describe the expression results of GPVI. The internal negative control for PE fluorescence intensity in each sample was used for normalization of GPVI expression as a means of standardizing patient results, which allows for effective comparative analysis.

Measurement of soluble GPVI levels was done using Human Soluble glycoprotein VI (sGPVI) ELISA kit supplied by Bioassay Technology Laboratory, Shanghai Korain Biotech Co., Ltd (Shanghai, China) as previously described [26, 27].

### Follow-up and endpoints

A monthly visit was done for all enrolled patients throughout the 12 months follow-up period of the study for safety and efficacy. Any adverse events were recorded; an increased liver function tests ≥ 3 times the upper normal level (ULN) or the kidney function tests above ULN, the occurrence of severe breakthrough bleeding with the need of rescue treatment, or a positive reticulin stain in the bone marrow ≥ grade 3 at 6 months of therapy were considered safety endpoints.

Outcome measures were percentage of patients achieving increased expression of glycoprotein VI collagen receptor, percentage of patients achieving complete response (CR) defined as any platelet count of 100 × 10^9^/L at least once throughout the study period in the absence of rescue treatment [19], percentage of patients achieving response (R) defined as any platelet count between 50 and 100 × 10^9^/L or doubling of the baseline count at least once throughout the study period in the absence of rescue treatment [19, 28] and the maximum duration for which a patient continuously maintained a platelet count between 50 and 100 × 10^9^/L in the absence of rescue treatment.

### Statistical analysis

Statistical analysis was done through SPSS software version 27 (IBM SPSS Statistics, IBM Corporation, Chicago, IL, USA). Kolmogrov-Smirnov test was used to examine the normal distribution of variables. Quantitative variables were described in the form of mean and standard deviation or median and interquartile range (IQR: 25th-75th percentiles). Qualitative variables were described as number and percent. To detect differences between the intervention and control groups, we used independent t test for quantitative parametric data while data with non-parametric distribution were analyzed using Mann-Whitey test. For comparison of categorical variables, the chi-square test was used. To determine the effects eltrombopag therapy on bleeding score, quality of life and hematological parameters, Analysis of covariance (ANCOVA) was performed to compare mean values between groups adjusted for differences in baseline measures of age, BMI, and laboratory variables. Variables which were not normally distributed were log-transformed before entering the analysis.

To identify within-group changes, we applied paired-samples t tests for quantitative parametric data measured at two time points while Repeated Measures Analysis of variance (ANOVA) followed by post hoc analysis using Bonferoni was used for variables measured at three time points. For non-paramateric data, Wilcoxon rank-sum test was used for data with non-parametric distribution measured at two time points while Friedman test followed by post hoc using Wilcoxon test was used for variables measured at three time points. To test the difference between paired proportions (i.e. the difference in the percentage of patients at three time points), the McNemar test was applied.

Pearson correlation coefficients were used to assess the association between two normally distributed variables. When a variable was not normally distributed, a Spearman correlation test was performed. Multivariable linear regression analysis was employed to determine the relation between GPVI and clinicopathological variables. A P value < 0.05 was considered significant in all analyses.

## Results

### Baseline clinical and laboratory characteristics of the studied population

The study included 36 children and adolescents with ITP (18 males and 18 females with a male to female ratio 1:1). The median (IQR) age of all patients was 10 (7–13.5 years). Two patients in the eltrombopag group dropped out; one of them had elevated liver function and stopped treatment.

No significant difference was found as regards baseline clinical or laboratory data between ITP patients with and without Eltrombopag therapy. The number of lines of therapy before the start of the study was similar between both groups (Table 1).

**Table 1:** Clinical and laboratory data among patients with ITP receiving eltrombopag therapy or not at baseline and at 6 months

Variable	Eltrombopag				Control				p-value^a^
	Baseline (n = 18)	At 6 months (n = 16)	Change	p-value^b^	Baseline (n = 18)	At 6 months (n = 18)	Change	p-value^b^	
**Age** (years)	8 (4–13)	-	-	-	10 (8–14)	-	-	-	0.119*
**Males**, n (%)	9 (50)	-	-	-	9 (50)	-	-	-	1.000†
**BMI SDS**	0.75 (0.2–1)	-	-		0.9 (0.8–1)	-	-		0.121*
**Bleeding score**	6 (5–7)	2 (0–5)	-60 (-100 – -31.0)	< 0.001	5.5 (4–6)	5 (4–6)	0 (-25.0–16.7)	0.209	0.021
**Skin bleeding**	18 (100)	9 (56.2)	-	0.015	18 (100)	16 (88.9)	-	0.500	0.031†
**Gum or oral mucosal bleeding**, n (%)	18 (100)	8 (50)	-	0.016	17 (94.4)	15 (83.3)	-	0.250	0.038†
**Epistaxis**, n (%)	10 (55.6)	4 (25)	-	0.063	9 (50.0)	6 (33.3)	-	0.453	0.595†
**Menorrhagia**, n (%)	4 (22.2)	0 (0)	-	0.250	4 (22.2)	2 (20.0)	-	0.500	0.207†
**Quality of life**	83.1 ± 2.7	89.2 ± 4.6	8.54 (6.56–10.1)	< 0.001	84.1 ± 1.5	84.5 ± 3.2	0 (-2.4–2.4)	0.635	0.002
**Loss of CR or R**^#^, n (%)	-	5 (45.5)	-	-	-	8 (88.9)	-	-	0.043
**Platelets** (x 10^9^/L)	5 (2–14)	48 (20– 93)	750 (354.8–1250)	< 0.001	11.5 (7–19)	23 (16–38)	106.8 (21.1 - 400)	0.004	0.028
**WBCs count**	6.98 ± 1.67	7.03 ± 1.01	9.2 (-23.3–23.1)	1.000	7.49 ± 1.19	7.01 ± 0.98	-4.6 (-17.1–7.89)	0.427	0.955
**BM reticulin stain grading**, n (%) Grade 0 Grade 1 Grade 2	7 (38.9) 11 (61.1) 0 (0.0)	3 (18.8) 11 (68.8) 2 (12.5)	-	0.116	9 (50) 9 (50) 0 (0)	9 (50) 9 (50) 0 (0)	-	1.000	0.078
**GPVI flow cytometry (Normalized median)**	1.88 ± 0.49	2.94 ± 0.94	47.9 (4.61–138.2)	0.008	1.83 ± 0.42	1.73 ± 0.50	-7.9 (-26.2–6.1)	0.715	0.003
**Soluble GPVI** (ng/mL)	12 (9–18)	33 (23–63)	213.6 (68.8–295.7)	0.002	8 (3.0–15.5)	2.5 (2.0–3.0)	-56.3 (-86.9–0.0)	0.043	< 0.001

SDS: standard deviation score; BMI: body mass index; CR: complete response; R: response; WBCs: white blood cells; BM: bone marrow; GPVI: glycoprotein VI

^#^Relapse (Loss of CR or R) was calculated in patients who achieved CR

^a^P value was obtained using Analysis of covariance (ANCOVA) unless specified (*: Mann-Whitney test was used; †: Chi-square test was applied)

^b^P value was obtained from paired-samples t tests for parametric variables, Wilcoxon rank-sum test for non-parametric variables or Chi-square test for categorical variables

### Effect of eltrombopag therapy on bleeding score, quality of life, platelet count, GPVI expression and soluble levels

Baseline GPVI expression levels by flow cytometry and soluble GPVI levels were significantly lower among all the studied ITP patients compared with healthy controls; 1.86 ± 0.45 versus 4.8 ± 1.1 and 11 (8–18) ng/mL versus 82 (71–97) ng/mL, respectively; p = 0.001).

**Fig. 1:**
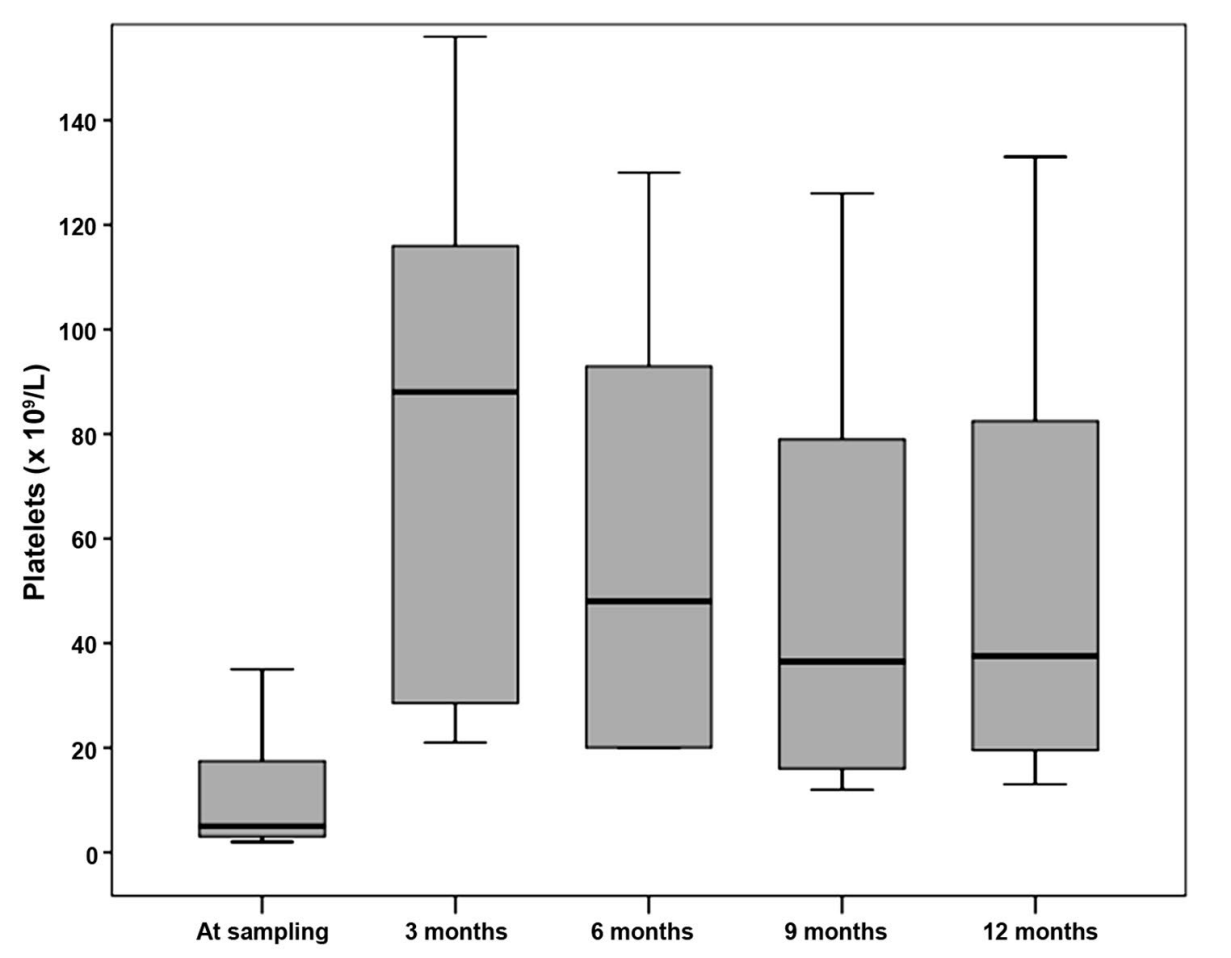
Platelets count among ITP patients who received eltrombopag therapy throughout the study period

As shown in Table 1, bleeding score represented in skin, gum bleeding, epistaxis and menorrhagia was significantly decreased in ITP patients who received eltrombopag after 6 months of therapy while the quality of life had significantly improved compared to baseline levels or to those who did not received eltrombopag. Platelet count was significantly increased throughout the study after eltrombopag therapy (Fig. 1). Moreover, GPVI expression by flow cytometry and soluble GPVI level were significantly increased after therapy in the intervention group compared to the control group. It is important to note that bone marrow reticulin stain showed no significant difference before and after eltrombopag therapy or compared with patients who did not receive eltrombopag. The number patients who lost CR or R was significantly lower among eltrombopag group than the control group. On the other hand, ITP patients who did not receive eltrombopag therapy showed no significant difference as regards the studied variables at baseline, 3 months and 6 months.

Further follow-up of ITP patients who received eltrombopag therapy at 9 months and 12 months after therapy (Table 2) showed that those patients were able to maintain a low bleeding score and a good quality of life throughout the follow-up period. Moreover, patients who received eltrombopag therapy were able to maintain the platelet count achieved at 6 months till 12 months although it was mildly decreased but without any significant difference (Fig. 1).

**Table 2:** Clinical and laboratory data of ITP patients who received eltrombopag at 6 months, 9 months and 12 months after therapy

Variables	6 months (n = 16)	9 months (n = 16)	12 months (n = 16)	p value			
				Overall	P1	P2	P3
**Bleeding score** median ± SD	2 (0–5)	2.5 (0–4.5)	2.5 (0–4.5)	0.819^≠^	1.000	1.000	1.000
Skin bleeding*, n (%)	9 (56.2)	10 (62.5)	10 (62.5)	**--**	1.000	1.000	1.000
Gum or oral mucosal bleeding*, n (%)	8 (50.0)	8 (50.0)	8 (50.0)	**--**	1.000	1.000	1.000
Epistaxis*, n (%)	4 (25.0)	4 (25.0)	4 (25.0)	**--**	1.000	1.000	1.000
Menorrhagia*, n (%)	0 (0.0)	1 (14.3)	1 (14.3)	**--**	1.000	1.000	1.000
**Quality of life**, mean ± SD	89.19 ± 4.64	**---**	89.06 ± 5.58	0.795•	**--**	0.795	--
**Platelets** (x 10^9^/L), median (IQR)	48 (20– 93)	36.5 (16–79)	38 (19.5–83)	0.368^≠^	0.178	0.426	0.114
**WBCs count** (x 10^9^/L), mean ± SD	7.03 ± 1.01	6.83 ± 0.98	7.08 ± 0.86	0.627^••^	1.000	1.000	0.613

WBCs: White blood cells

^≠^: Friedman test was used followed by post hoc using Wilcoxon test; *: McNnemar test was used; ^•^: Paired t-test was used; ^••^: p value was obtained from Repeated Measures Analysis of covariance (ANOVA) followed by post hoc analysis using Bonferoni

P1: Disease characteristics at 6 months versus 9 months

P2: Disease characteristics at 6 months versus 12 months

P3: Disease characteristics at 9 months versus 12 months.

Eleven ITP patients achieved CR while none of the patients showed a response (R) as per definition. No significant difference was found as regards bleeding score and quality of life between ITP patients who achieved CR after eltrombopag therapy at 6 months, 9 months and 12 months after therapy while platelet count showed a mild fluctuation that did not affect bleeding score or quality of life (p > 0.05).

Comparison of disease characteristics and laboratory data between ITP patients who received eltrombopag with NR at baseline and after 6 months (Table 3) showed a significant decrease in bleeding score and a significantly higher platelet count, GPVI expression and soluble GPVI.

There was a significant negative correlation between baseline GPVI expression by flow cytometry and bleeding score. Baseline soluble GPVI levels were positively correlated to platelet count while negatively correlated to bleeding score and WBCs count (Table 4). Multivariate linear regression analysis revealed that platelet count and GPVI expression by flow cytometry were the significant independent factors that affect soluble GPVI levels.

**Table 3:** Disease characteristics and laboratory data of ITP patients who received eltrombopag with no response (NR) at baseline and at 6 months

Variables	Baseline (n = 5)	6 months (n = 5)	p value
**Bleeding score**			
Median (IQR)	8 (6–8)	6 (4–7)	0.041
Range	5–9	2–8	
**Quality of life**			
Mean ± SD	82.2 ± 1.79	86.6 ± 5.68	0.198
Range	80–84	81–94	
**Platelets** (x 10^9^/L)			
Median (IQR)	2 (2–4)	25 (20–26)	0.043
Range	2–4	20–28	
**GPVI flow cytometry (Normalized median)**			
Median (IQR)	1.83 ± 0.41	2.7 ± 0.54	0.031
Range	1.52–2.17	1.89–3.62	
**Soluble GPVI** (ng/ml)			
Median (IQR)	11 (9–11)	36 (31–63)	0.043
Range	5–13	23–84	

**Table 4:** Correlation between soluble GPVI and the studied variables among all the studied ITP patients at baseline

Variables	Soluble GPVI at sampling	
	r	p value
**Age** (years)	0.012	0.955
**Weight SDS**	-0.109	0.595
**Height SDS**	0.168	0.413
**BMI SDS**	0.062	0.765
**Disease duration** (months)	-0.254	0.211
**Bleeding score at sampling**	-0.473	0.015
**Quality of life at sampling**	0.384	0.053
**Hemoglobin at sampling** (g/dL)	0.245	0.227
**Platelets at sampling** (x 10^9^/L)	0.556	0.008
**WBCs count at sampling** (x 10^9^/L)	-0.488	0.011
**GPVI flow cytometry (Normalized median)**	0.579	0.006

SDS: standard deviation score; WBCs; white blood cells.

## Discussion

The American Society of Hematology 2019 guidelines recommend that ITP children who do not respond to first-line therapies or who have chronic ITP should be trialed on TPO-RAs before rituximab and splenectomy [20]. Eltrombopag was the first TPO-RAs authorized for treatment of pediatric ITP [29].

In our study, there was no significant difference as regards platelet count between the intervention and control groups at the start of the study while at 6 months, platelet count was significantly higher among ITP patients treated with eltrombopag with a percentage of 68.8% (11 out of 16) of patients achieved count of 100 × 10^9^/L or more throughout the study. Furthermore, platelet count was significantly increased among non-responder patients after 6 months of eltrombopag therapy. The efficacy and safety of eltrombopag was assessed in children in the PETIT trials, which assessed pediatric patients with ITP [21, 28]. In PETIT2 trial which was a two part, randomized, multicenter, placebo-controlled study done at 38 centers in 12 countries, 40% of ITP children treated with eltrombopag achieved a platelet count of at least 50 × 10^9^/L without rescue therapy for 6 weeks or more from weeks 5 to 12 with a significantly greater proportion of patients in the eltrombopag group having a platelet response compared to patients in the control group [28].

In China, Chen and colleagues [30] showed that 83.3% of the patients achieving a platelet count of over 50 × 10^9^/L at least once in the absence of rescue therapy with a median platelet count of 78 (20–310 × 10^9^/L) at the end of their study. Moreover, Giordano et al. [29] revealed that at 6 months of eltrombopag treatment, the median platelet count was 92 (2–863 × 10^9^/L) and 68% of their ITP patients achieved a platelet count of at least 30 × 10^9^/L and 44% achieved a platelet count of at least 100 × 10^9^/L.

The quality of life is significantly affected in chronic ITP patients with prolonged disease duration [31]. Therefore, we used KIT scores to assess the quality of life. We demonstrated a significant improvement of the KIT scores in the group treated with eltrombopag at 6 months compared to control group. Similarly, Grace et al. [32] showed a significant improvement in the KIT score among their ITP patients. However, in PETIT study analysis for the health-related quality of life [33], there was a wide variability in KIT total scores at baseline and an insignificant change over time during treatment, even when considering only responders.

The platelet collagen receptor, GPVI is a member of the Ig receptor family, it is homologous to immune receptors and its engagement leads to platelet aggregation [34]. GPVI is essentially all uncleaved on normal circulating platelets, but is shed from the platelet surface in a metalloproteinase-dependent manner in response to GPVI ligands (including collagen) [35]. Changes in the surface levels of GPVI can be quantified by fluorescence-activated cell sorting (FACS) and/or shed soluble GPVI in plasma by ELISA or bead-based immunoassay [36]. The occurrence of anti-GPVI autoantibodies in ITP has been previously reported which may explain the observed depletion of GPVI from the platelet surface in ITP patients [37].

Interestingly, GPVI levels increased significantly after treatment with eltrombopag for 6 months, both by flow cytometry and ELISA. Furthermore, when comparing the intervention and control groups, patients who received eltombopag showed a significant increase in GPVI expression by flow cytometry and soluble GPVI levels with a significant decrease in bleeding score. This could be attributed to the concomitant increase in platelet count after 6 months of eltrombopag therapy especially that multivariate linear regression analysis revealed that platelet count was a significant independent variable affecting soluble GPVI levels. On the other hand, among eltrombopag non-responders, there was also a significant increase in the GPVI expression and soluble GPVI levels as well as decrease in bleeding score after 6 months of treatment.

To our knowledge, the effect of eltrombopag therapy on platelet GPVI has not been studied in pediatric ITP patients. However, in adults with ITP, Chiou and colleagues [38] conducted an open-label, multicenter study in which 25 Taiwanese patients with chronic ITP and investigated the levels of GPVI on the platelet surface by flow cytometry among those who received eltrombopag at baseline and after 6 months. They found that the percentage of bleeding patients was significantly reduced in both responders and nonresponders by 50% from the baseline level throughout the treatment period. Moreover, the mean fluorescence intensity (MFI) of GPVI escalated gradually after the administration of eltrombopag in responders. Also, the expression of GPVI was remarkably promoted in the UT-7/TPO megakaryoblastic cell line in a dose-dependent manner after eltrombopag treatment [38] probably via FLI1-mediated mechanism [13]. The authors concluded that GPVI expression was significantly up-regulated after treatment with eltrombopag and reported that low-to-intermediate dose of eltrombopag showed good efficacy to expedite platelet production and augment platelet adhesion. These two factors might explain the efficacy of eltrombopag in ameliorating hemorrhage in patients with ITP [38].

In this regard, Haselboeck et al. [39] showed an increase in P-selectin and in platelets monocytes aggregates among ITP patients on eltrombopag which indicate ongoing platelet activation during the early period of treatment. In contrast, Psaila et al. [10] investigated the effect of eltrombopag on platelets function using platelet GPIb and activated GPIIb/IIIa, P-selectin expression and found that only a slight increase in P-selectin was observed as well as a slight increase in platelet reactivity to thrombin receptor activating peptide (TRAP) in eltrombopag responders. The authors concluded that eltrombopag did not cause platelet activation or hyper-reactivity, irrespective of whether the platelet count increased. Yet, patients treated with romiplostim, another TRAs which was shown to be successful in ITP treatment after standard treatment failure [4], showed changes in platelet function parameters with cessation of bleeding even without an increase in platelet count although these data was not sufficient to study the association of the platelet size and pre-activation changes on romiplostim with bleeding risks independently of platelet count [40]. Moreover, Kanaji and colleagues [14] showed that romiplostim treatment may directly up-regulate the expression of platelet GPVI through receptor-mediated demethylation of the GPVI promoter. Again, all published studies in the context of the effect of eltrombopag on platelets reactivity had limited sample size. In our study, eltrombopag did not affect the bone marrow reticulin stain in our studied patients which further emphasis its safety in persistent or chronic ITP children.

In conclusion, our data suggest that eltrombopag, through its role in up-regulation of GPVI receptor expression and increase soluble GPVI levels, might reduce bleeding manifestations, decrease bleeding score and improve the quality of life of chronic and persistent ITP children independent of its effect on platelet count suggesting the possible role of eltrombopag in enhancing platelet function. Eltrombopag was safe and well-tolerated. Additional studies are needed to further clarify and prove the effect of eltrombopag on the platelets functions.
